# Increasing colon cancer testing in rural Colorado: evaluation of the exposure to a community-based awareness campaign

**DOI:** 10.1186/1471-2458-9-288

**Published:** 2009-08-10

**Authors:** Linda Zittleman, Caroline Emsermann, Miriam Dickinson, Ned Norman, Kathy Winkelman, Grace Linn, John M Westfall

**Affiliations:** 1 Department of Family Medicine, University of Colorado School of Medicine, Aurora, Colorado, USA; 2 High Plains Research Network and High Plains Research Network Community Advisory Council, University of Colorado School of Medicine, Aurora, Colorado, USA; 3 Creative Media Solutions, Pine, Colorado, USA

## Abstract

**Background:**

Despite effective prevention and early detection screening methods, colorectal cancer is the second leading cause of cancer death in the United States. Colorectal cancer screening community-based interventions are rare, and the literature lacks information about community-based intervention processes. Using participatory research methods, the High Plains Research Network developed a community-based awareness and educational intervention to increase colorectal cancer screening rates in rural northeastern Colorado. This study describes the program components and implementation and explores whether the target population was exposed to the intervention, the reach of the individual intervention components, and the effect on screening intentions.

**Methods:**

A random digit dial survey was conducted of residents age 40 and older in the first 3 communities to receive the intervention to estimate exposure to the intervention and its effect on colorectal cancer screening intentions.

**Results:**

Exposure to at least intervention component was reported by 68% of respondents (n = 460). As the level of exposure increased, intentions to talk to a doctor about colorectal cancer screening increased significantly more in respondents who had not been tested in the past 5 years than those who had (p = .025). Intentions to get tested increased significantly in both groups at the same rate as level of exposure increased (p < .001).

**Conclusion:**

Using local community members led to the successful implementation of the intervention. Program materials and messages reached a high percentage of the target population and increased colorectal cancer screening intentions.

## Background

Despite effective prevention and early detection screening methods, colorectal cancer (CRC) is the 2nd leading cause of cancer death in the United States [1]. Unlike many other screening tools, such as mammography that detects existing cancer, CRC screening methods can prevent cancer from developing through the removal of pre-cancerous polyps. When CRC is found early and treated, the 5-year relative survival rate is 90 percent. However, less than 40% of colorectal cancers are found early due to the underutilization of CRC screening [2]. In 2005, only 47% of adults age 50 and older in the U.S. reported having either a fecal occult blood test within the past year, sigmoidoscopy within the past five years, or a colonoscopy within the past 10 years, as recommended in the United States by the Centers for Disease Control and Prevention (CDC) and the American Cancer Society [3]. An estimated 153,760 people in the U.S. were expected to be diagnosed with CRC in 2007, costing over $8 billion in treatment [4,5]. Of the 52,180 people who were expected to die from CRC in 2007 in the U.S., half may have been prevented if all people age 50 and older were screened regularly [1].

This study took place in rural and frontier counties which are part of the High Plains Research Network (HPRN) in eastern Colorado. In 2006, 30% of adults in the state of Colorado age 50 and older had an FOBT within the past 2 years, and 58% report ever having a proctoscopy, colonoscopy, or sigmoidoscopy [6]. In rural and frontier eastern Colorado, a recent survey by the authors of age-eligible residents reported similar use of FOBT (32%), but only 51% had ever had flexible sigmoidoscopy or colonoscopy testing. Almost a quarter (23%) of the rural sample had never had any type of CRC screening [7]. Colorectal cancer screening remains an underutilized method of cancer prevention and early detection, and rural areas may be susceptible to even lower screening rates.

Compared to other cancers such as breast and cervical, community-based interventions targeting CRC screening are rare [8]. The Center for Disease Control and Prevention's (CDC) community preventive guidelines include no evidence-based recommendations for effective community interventions to improve CRC screening [9]. In an attempt to fill this gap, the HPRN formed a group of local residents from eastern Colorado to develop a community-based CRC awareness and educational campaign uniquely tailored to a rural population with the goal of increasing colorectal cancer screening rates in residents age 50 and older in northeast Colorado. This paper describes the intervention implementation, reach of the materials, and the effect exposure to the intervention had on screening intentions in the rural target population. The study was conducted in the first 3 communities to receive the intervention and was funded by the CDC.

### The Study Region: High Plains Research Network

The HPRN is a practice-based research network that covers the 16 counties of eastern Colorado. Of these counties, 10 are designated "frontier" (or less than 6 people per square mile), and the remaining 6 are "rural". The entire HPRN consists of 55 primary care practices, 16 hospitals, approximately 150 providers, and the communities that reside in this region. The 3 communities involved with this particular evaluation included 2 smaller communities (populations provided below), each with 1 primary care practice each. The third town is the largest community in the area and is home to 6 practices, including private solo practices and a hospital-based clinic. Ranching and farming are dominant sources of income and permeate the culture of this region. Residents living in the 3 counties included in this evaluation are older than the state as a whole, with a median age of 39 versus 34 for the state and a percent of the population age 65 and older of 16% versus 10% [10].

### The Intervention: Testing to Prevent Colon Cancer in Rural Colorado

The HPRN launched a community-based, multi-component colon cancer prevention intervention in the spring of 2006. "Testing to Prevent Colon Cancer in Rural Colorado" aims to increase CRC screening behaviors, knowledge, and attitudes in rural northeastern Colorado. The intervention consisted of an awareness and educational campaign that encouraged local residents to talk to their providers about colon cancer testing options, targeting residents age 50 and older.

Community-based participatory research (CBPR) methods were used to develop, implement, and test the intervention messages and materials. While the definition of community may vary by project, CBPR research involves a collaborative partnership *with *a group to increase the relevance of the research to the community. The HPRN uses CBPR approach regularly, having formed a Community Advisory Council (C.A.C.) in 2003 to help to guide the research it conducts. The C.A.C. consists of 9 residents of rural northeast Colorado, including farmers, teachers, ranchers, a home health visitor, and a retired administrator. Specifically for this intervention, the C.A.C. was expanded to include 2 local physicians, 2 public health workers, and a hospital administrator from northeast Colorado. The resulting group, named the Joint Planning Committee (JPC), designed the intervention's main messages, materials, and implementation strategies.

The JPC developed a 4-point message to address key catalysts of behavior change, including relevance, education, facilitation/encouragement, and action. The main messages are below.

1. Colon cancer is the 2^nd ^leading cause of cancer death in the U.S.

2. Colon cancer is preventable.

3. Testing is worth it.

4. Talk to your doctor today.

### Implementation Evaluation

The program's 4 main messages were incorporated into the intervention's materials, a set of 8 mostly bilingual components. Table 1 provides a description of each component and implementation. The JPC selected program components that could be implemented in each intervention community and that linked community members back to their local primary care providers. Methods were selected that tapped into the communication culture of rural communities, including the use of local community members in program components, local newspapers and adaptations of familiar small print materials (communication methods engrained into every day life in rural towns), and local organizations that are very common and valued in rural communities. The JPC helped identify local residents to have their photos taken for the palm card series and series of ads in the local newspapers, to share their personal stories in local newspapers, and to co-present community talks with a local health care provider. All local newspapers agreed to run the 3 newspaper components of the intervention. Local residents and clinic staff were also recruited to distribute the small print materials, such as the palm cards and farm auction flyers, and recorded the number of copies left at each location. All primary care practices in the intervention communities agreed to distribute the "got polyps?" travel mugs to residents who redeemed a palm card or newspaper ad for a mug at the clinic.

**Table 1:** Implementation of "Testing to Prevent Colon Cancer in Rural Colorado" in Three Communities

**Component**	**Description**	**Dissemination**
Palm Card	Postcard-sized "palm cards" with photos of local residents and the program's 4 main messages. Double-sided. English and Spanish.	2300 cards placed at 45 locations, such as coffee shops, pharmacies, livestock auction businesses, farm equipment and implement dealers, hardware stores, lumberyards, feed stores, barbershops, golf shops, flower shops, auto parts stores, libraries, and liquor stores

Farm Auction Flyer	Specifically designed to target the hard-to-reach male farmers. Describes colon cancer prevention. Masqueraded as a commonly seen document that is often quickly grabbed for further study.	Hung or left on countertops in stacks at 71 locations frequented by the agricultural population and anywhere regular farm auction flyers are normally hung. See examples of locations above.

Posters	Displays key messages with photos of local residents. English and Spanish.	7 hung at 7 locations

Community Talks	PowerPoint presentation on CRC prevalence and screening methods. Co-presented by local physicians and community members. Speakers were paid. English and Spanish.	13 talks (3–6 per town) attended by 265 people

"got polyps?" Mugs	Stainless steel travel mug that reads "got polyps?" in English and "Polyps? Prevent them, don't get them!" in Spanish. Handed out at local clinics to people redeeming a palm card.	480 given out to patients at 8 clinics

Personal Stories	A series of personal ("human interest") stories in the newspapers about local residents and CRC SCREENING; 1 story per week for 4 weeks. A larger community printed 8 stories between 2 newspapers.	16 printed in 4 newspapers (4 per paper)

Ads	Newspaper ads with photos of local residents and the 4 program messages; 1 ad per week for 4 weeks. A larger town printed 10 ads between 2 newspapers.	17 ads ran in 4 newspapers (4–5 per paper)

Medical Articles	A 4-part series of newspaper articles about the medical facts of colon cancer; 1 article printed per week for 4 weeks.	same 4 articles ran in each paper

## Methods

This study was conducted in the first 3 communities to receive the intervention and the farmland counties in which they reside: Julesburg (population estimates: zip code = 1818; age ≥ 50 = 721); Haxtun (population estimates: zip code = 1608; age ≥ 50 = 622), and Sterling (population estimates: zip code = 16,486; age ≥ 50 = 4,695) [10].

### Survey Design

A random digit dial (RDD) telephone survey was conducted to estimate exposure to the community-based CRC prevention intervention in the target population and to determine if exposure increased intentions to talk to a doctor about CRC screening and to get tested.

The survey was conducted with a stratified random sample of residents living in the first 3 communities and surrounding areas to receive the intervention 3 months after the intervention had been implemented. Participants were sampled by age group (40 – 49 years of age and ≥ 50 years of age) and stratified by intervention community using zip codes. Two towns are substantially smaller than the third. The smaller towns' populations were over-sampled to produce more reliable estimates.

A Colorado market research firm conducted interviews for 1 week in July 2006 using 3 sample lists from Survey Sampling International: 1) household phone numbers from 3 communities having at least 1 adult age 50 or older, 2) household phone numbers from 3 communities having at least 1 adult age 40–49, and 3) household phone numbers for respondents living in surrounding areas within each county with at least 1 adult age 40 or older. Respondents aged 40–49 were included in this study since they are approaching the screening-eligible age of 50 or could be eligible for screening based on family history. A small sample from the surrounding areas was included to provide preliminary estimates of the reach of the intervention outside of the 3 primary communities. Age-eligible respondents with the most recent birthday were selected within each household.

Information about exposure to the intervention during the previous 3 months was collected. Exposure questions included both recall and recognition. Respondents were first asked to freely recall sources of colon cancer prevention information that they had seen, heard, or read in the past 3 months (recall). Respondents were then read a description of each intervention component not freely recalled and asked if they had seen, heard, or read the component (recognition). To estimate the depth of exposure to individual components, the number of exposures to each component was also recorded.

Respondents reporting exposure to any type of colon cancer prevention information were asked questions about intended behavior change as a result of this information. A 5-point Likert scale assessed how likely respondents were to talk to their doctors about CRC screening and how likely they were to get screened. Demographic questions included age, gender, ethnicity, and race and if they had been tested for colon cancer in the past 5 years. This question did not specify type of screening procedure. A copy of the survey is available at: .

### Statistical Analysis

Descriptive analyses were performed for the demographic variables age, gender, race and ethnicity, geographic location, and having been tested for colon cancer in the past 5 years. Chi-squared tests were performed to determine general associations between the demographic variables and exposure ("exposed" and "not exposed"). Respondents recalling or recognizing at least one of the 8 intervention components were considered exposed. Respondents who did not report any sources of colon cancer information or who exclusively reported CRC screening materials other than our 8 intervention components were considered "not exposed". Frequencies were run on the number of personal stories, newspaper ads, and medical articles read. Total and individual intervention component exposure rates were calculated.

A general linear model was performed to determine multivariate associations between exposure to the intervention and intended CRC screening behaviors. CRC screening behaviors included the likelihood to talk with a doctor about being tested and the likelihood to get tested for colon cancer. CRC screening behaviors were run as a continuous outcome variable, ranging from 1 = "strongly disagree" to 5 = "strongly agree". Exposure was defined by an ordinal aggregate variable ranging from 0 (exclusively reported CRC screening materials other than the intervention components) to 6 (reported at least 6 intervention components) and is referred to as "dose". Since the components were designed to be implemented as a whole intervention, this analysis measured the effectiveness of the intervention collectively. Models adjusted for age, gender, past 5-year testing status, race and ethnicity, and intervention dose. To adjust for over-sampling, responses were weighted by the inverse of the sampling fraction of each town. Weighted estimates are reported. Interaction between intervention dose and past 5-year testing status was also adjusted in each model. Prior to the final analysis, the ordinal exposure variable was tested for linear, quadratic, and cubic trends for each outcome. A p-value of < .05 was considered statistically significant.

All analyses were performed using SAS version 9.1. The survey instrument and methodology was approved by the Combined Institutional Review Board at the University of Colorado Denver and Health Sciences Center.

## Results

A total of 460 residents from the first three intervention communities (n = 400) and their surrounding areas in each county (n = 60) completed the survey. The response rate for households in which an eligible respondent was identified and contacted (n = 1032) was 45%, comparable to the response rate of the 2005 Behavioral Risk Factor Surveillance Survey [11]. Table 2 summarizes the demographic characteristics of the survey sample. Of the 460 respondents, 47% were female; 55% were age 50 or older; 88% were White, non-Hispanic; and 54% reported having had no CRC screening in the past 5 years.

**Table 2:** Demographics Characteristics of Respondents, N = 460

	Unweighted N = 460	Unweighted %	Weighted %
**Age**			

40–49	209	45	40

50–64	109	23	28

≥ 65	142	31	32

**Gender**			

Male	244	53	54

Female	216	47	46

**Race/Ethnicity**			

White, non-Hispanic	403	88	86

Other	57	12	14

**Tested in Past 5 Years**			

Yes	210	46	47

No	250	54	53

### Exposure to Intervention

**Figure 1:**
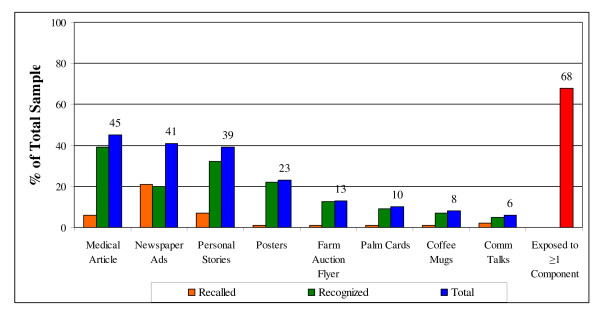
**Exposure by Component and Overall (n = 460)**.

Of the 460 respondents, 68% reported exposure to at least one intervention component. Females were more likely to report exposure to the intervention materials than were males (74% vs. 63%, p = .02). Respondents who reported having been tested in the past 5 years were more likely than those not reporting testing in the past 5 years to have been exposed to the materials (78% vs. 58%, p < .001). The percent of the total respondents reporting exposure to each intervention component is displayed in Figure 1. The 3 newspaper components exhibited the most reach.

Based on these results, additional analysis was conducted to determine how many respondents saw only the newspaper component(s), a combination of components, or only non-newspaper components (Table 3). Of the total sample, 30% read at least one newspaper component only, 30% saw some combination of newspaper and other intervention components, and 8% saw other intervention components only. Overall, 60% of the sample was exposed to at least one of the newspaper components, and 38% saw at least one of the small-scale print materials or community talk.

**Table 3:** Reported Exposure: Mass Media vs. Other Component Type

**Type of Intervention Exposure**	**# of Respondents**	**% Total Sample** **(n = 460)**	**% Exposed Sample** **(n = 311)**
Newspaper Only (≥ 1 Newspaper Component Exclusively)	126	30	41

Combination (≥ 1 Newspaper Component and Other	146	30	47

Other Intervention Components Only (Saw Other Components Only)	39	8	13

Not Exposed to Any Intervention Components	149	32	na

TOTAL	460	100	100

Among respondents exposed to the intervention, the average number of unique components recalled or recognized was 2.8. Of the total sample, only 32% reported no exposures. Comparable proportions of respondents were exposed to 1, 2, 3, and 4 components (17%, 16%, 13%, and 12% respectively). Approximately 8% of the respondents reported exposure to 5–8 intervention components.

### Intentions for CRC Screening

**Figure 2:**
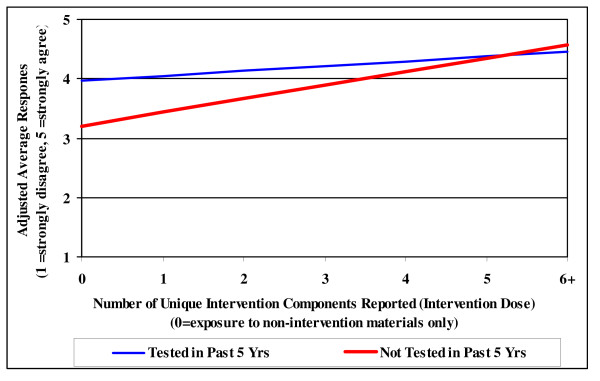
**Intentions to Talk to Doctor by Testing Status and Intervention Dose (n = 369)**.

**Figure 3:**
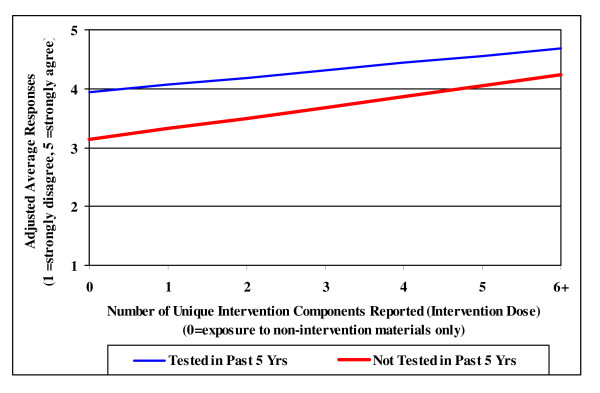
**Intentions to Get Tested by CRC Testing Status and Intervention Dose (n = 369)**.

Multivariate analyses determined an increasing linear trend between the number of intervention items seen and the intent to "talk to a doctor about colon cancer testing" and to "get tested". Figures 2 and 3 display the adjusted rate of increase for each outcome by intervention dose. Results for intentions did not differ by age, gender, or race, including those respondents age 40–49. Respondents who had not been tested in the past 5 years reported lower intentions to talk to a doctor about colon cancer and to get tested for colon cancer than their tested counterparts at low intervention doses. However, as intervention dose increased, intention to talk to a doctor about colon cancer increased at a significantly faster rate in respondents who had not been tested than in those who had been tested in the past 5 years (p = .025; Figure 2). As shown in Figure 3, respondents who had not been tested in the past 5 years reported overall lower intentions to get tested compared with those who had been tested (p < .001). However, intentions to get tested increased at a significantly similar rate for both groups (p < .001).

## Discussion

Using a community-based participatory research method, the "Testing to Prevent Colon Cancer in Rural Colorado" intervention was extremely successful at reaching its target population in the studied region, with 68% of the study sample reporting exposure to one or more of the intervention components. Other population-based health interventions report exposure levels range from 14% to 71% [12-16]. Two large-scale commercial CRC campaigns also report reaching high percents of their target populations. A 1-year television advertising strategy co-sponsored by the American Cancer Society used donated broadcast time valued at $21.3 million and reported reaching 40% of target households [17]. Utah Cancer Action Network's state-wide campaign used donated television, radio, and print airtime and inserts along with grassroots efforts and reached 79% of survey respondents [18]. "Testing to Prevent Colon Cancer in Rural Colorado" reached a comparable level of its target population using fewer forms of mass communication strategies and spending approximately $26, 000 on consultation and component production for the first 3 towns. From another perspective, our target population was as aware of this CRC intervention as typical American adults are aware of the popular culture phenomenon Harry Potter (69%) [19].

A second key finding is the positive effect of the intervention on respondents' intentions to talk to a doctor about CRC screening and to get tested. This is an important finding as intention to undertake a behavior has been shown to be a strong predictor of actual behavior [20,21]. This program effectively drove home its action message "talk to your doctor today", strongly influencing respondents who had *not *been tested in the past 5 years by increasing their intent to talk to a doctor about CRC screening. This is important because a frequently reported barrier to screening is a lack of both information about the tests and physician recommendation for a test, while a strong predictor of screening is the patient asking for a test [7,22-24]. Additionally, this campaign significantly increased intentions to get tested at the same rate in both "tested" and "not tested" groups. Increases in both measures of intentions did not differ by age. Since this study included residents age 40–49, the intervention demonstrated a positive influence on respondents who are less likely to have been previously tested and who are at important pre-contemplative age. These results are encouraging for attempts to attain or maintain patients' status of being up-to-date on CRC screening.

This study illustrates the advantages of using multiple components in a rural setting to maximize both reach and behavioral intentions. None of the individual components resulted in the same level of exposure as the overall intervention. The small-scale print materials and community talks increased the reach of this intervention by nearly 10%. Of the 60% of respondents reporting exposure to at least one newspaper component, half of them increased their intervention dose by at least 1 as a result of additional exposures to non-newspaper components. The significant positive effect of intervention dose on intentions emphasizes this point.

Partnering with local community members was vital to development and implementation this intervention, which in turn effects reach and effectiveness. Having community members craft the messages and methods, take control of disseminating small print materials, and present community talks ensured the feel of the materials would be appropriate for the audience and placed to be most effective.

### Limitations

Limitations of this study include the use of self-reported data. Although the standard method for this type of community-based survey, some error in product recall or recognition may have occurred. We cannot assess exposure in people who do not have access to a phone or who use cell phones as their household phone. However, we estimate that more than 90% of the target population in this region has a landline telephone. Due to the nature of random digit dial telephone surveys, we do not have information about non-responders and are unable to determine how results may be biased as a result. This intervention was designed to be administered as a whole unit, with pieces complimenting each other or reaching certain groups. Thus, we did not analyze the effect of individual components on CRC screening intentions. Finally, while the intervention materials were largely bilingual, this phone survey was conducted with English-speakers only. However, the proportion of Spanish-speaking only community members in our targeted age range is small. The vast majority of Spanish-speaking only people in this region are under age 40.

## Conclusion

The use of participatory research methods resulted in a culturally relevant community-based CRC intervention that can be successfully implemented in rural communities. The multi-component intervention effectively reached and positively changed CRC screening intentions in its target population.

## Competing interests

GL owns a private media development and consultation company. GL was a paid consultant, working on the design and production of the intervention print materials.

The remaining authors declare that they have no competing interests.

## Authors' contributions

JW, LZ, CE, and MD were responsible for the conception of the study, study design, data collection, and processing. LZ coordinated data collection and drafted the manuscript. CE drafted the analysis plan and performed the statistical analysis. NN and KW assisted with drafting the manuscript with specific focus on the description of the intervention and implementation. GL assisted with the draft of the manuscript with specific focus on the description of the intervention materials. JW, LZ, CE, MD, NN, KW, and GL were all involved with data interpretation and critical revisions of the paper. All authors provided approval for its publication.

## Pre-publication history

The pre-publication history for this paper can be accessed here:



## References

[B1] Cancer Prevention and Early Detection Facts and Figures American Cancer Society. http://www.cancer.org/downloads/STT/CPED2007PWSecuredCPED.pdf.

[B2] Colorectal Cancer Screening Rates Centers for Disease Control and Prevention. http://www.cdc.gov/cancer/colorectal/statistics/screening_rates.htm.

[B3] Cancer Prevention and Early Detection Facts and Figures American Cancer Society. http://www.cancer.org/downloads/STT/CPED_2008.pdf.

[B4] Ries LAG, Melbert D, Krapcho M, Mariotto A, Miller BA, Feuer EJ, Clegg L, Horner MJ, Howlader N, Eisner MP, Reichman M, Edwards BK, eds SEER Cancer Statistics Review, 1975–2004. National Cancer Institute. Based on November 2006 SEER data submission. http://seer.cancer.gov/statfacts/html/colorect.html?statfacts_page=colorect.html&x=11&y=15.

[B5] Brown ML, Riley GF, Schussler N, Etzioni RD (2002). Estimating health care costs related to cancer treatment from SEER-Medicare data. Med Care.

[B6] State Summary of BRFSS Data for 2004 Colorado Dept. of Public Health and Environment. http://www.cdphe.state.co.us/hs/brfss/HPCO06.pdf.

[B7] Young W, McGloin J, Zittleman L, West D, Westfall J (2007). Predictors of Colorectal Screening in Rural Colorado: Testing to Prevent Colon Cancer in the High Plains Research Network. J Rural Health.

[B8] Curbow B, Bowie J, Garza M, McDonnell K, Scott L, Coyne C, Chiappelli T (2004). Community-based cancer screening programs in older populations: making progress but can we do better?. Prev Med.

[B9] Cancer Guide to Community Preventive Services Website Centers for Disease Control and Prevention. http://www.thecommunityguide.org/cancer/.

[B10] US Census Fact Finder 2000 Census Data. http://factfinder.census.gov/home/saff/main.html?_lang=en.

[B11] Center for Disease Control and Prevention (2006). 2005 Behavioral Risk Factor Surveillance System Summary Data Quality Report. http://ftp.cdc.gov/pub/Data/Brfss/2005SummaryDataQualityReport.pdf.

[B12] Walls CT, Lauby J, Lavelle K, Derby T, Bond L (1998). Exposure to a community-level HIV prevention intervention: who gets the message?. Journal of Community Health.

[B13] Palmgreen P, Donohew L, Lorch EP, Hoyle R, Stephenson M (2001). Television Campaigns and Adolescent Marijuana Use: Tests of Sensation Seeking Targeting. Amer J Public Health.

[B14] Macarthur C (2003). Evaluation of Safe Kids Week 2001: prevention of scald and burn injuries in young children. Injury Prevention.

[B15] Vega MY, Roland EL (2005). Social marketing techniques for public health communication: a review of syphilis awareness campaigns in 8 US cities. Sex Transm Dis.

[B16] Wray RJ, Jupka K, Ludwig-Bell C (2005). A community-wide media campaign to promote walking in a Missouri town. Prev Chronic Dis.

[B17] ARF Study Shows PSA Impact Public Service Report. PSA Research Center.

[B18] Heins J, Broadwater C, Hoelscher C, Adam M, Cami R (2004). Skin and Colon Cancer Media Campaigns in Utah. Prev Chronic Dis.

[B19] Different Groups Follow Harry Potter (2001). The Barna Group of Ventura California. http://www.barna.org.

[B20] Webb T, Sheeran P (2006). Does Changing Behavioral Intentions Engender Behavior Change? A Meta-Analysis of the Experimental Evidence. Psychological Bulletin.

[B21] Ajzen I (1991). The Theory of Planned Behavior. Organizational Behavior and Human Decision Process.

[B22] Seeff LC, Nadel MR, Klabunde CN, Thompson T, Shapiro JA, Vernon SW, Coates RJ (2004). Patterns and predictors of colorectal cancer test use in the adult U.S. population. Cancer.

[B23] Straus WL, Mansley EC, Gold KF, Wang Q, Reddy P, Pashos CL (2005). Colorectal Cancer Screening Attitudes and Practices in the General Population: A Risk-adjusted Survey. J Public Health Management Practice.

[B24] Levy BT, Dawson J, Hartz AJ, James PA (2006). Colorectal Cancer Testing Among Patients Cared for by Iowa Family Physicians. Amer J of Prev Med.

